# Effectiveness of a fortified drink in improving B vitamin biomarkers in older adults: a controlled intervention trial

**DOI:** 10.1186/s12986-021-00630-8

**Published:** 2021-12-07

**Authors:** Maria Heffernan, Leanne C. Doherty, Roberta Hack Mendes, Michelle Clarke, Stephanie Hodge, Michelle Clements, Liadhan McAnena, Mari Rivelsrud, Mary Ward, J. J. Strain, Helene McNulty, Lorraine Brennan

**Affiliations:** 1 UCD School of Agriculture and Food Science, Institute of Food and Health, Conway Institute, University College Dublin, Belfield, Dublin 4, Ireland; 2 Nutrition Innovation Centre for Food and Health (NICHE), School of Biomedical Sciences, Ulster University, Coleraine, Northern Ireland UK; 3 Smartfish AS, Oslo, Norway

**Keywords:** Folate, Folic acid, Vitamin B12, Vitamin B6, Riboflavin, Fortified drinks, B vitamin biomarkers, Older adults

## Abstract

**Background:**

Older adults are reported to have sub-optimal B vitamin status; targeted food-based solutions may help to address this. The objectives of the OptiAge food intervention study were to develop and investigate the effectiveness of a B vitamin-fortified drink in improving B vitamin biomarkers in older Irish adults with a primary outcome of change in the B vitamin biomarker status.

**Methods:**

A double-blinded randomised controlled trial was performed in parallel at University College Dublin and Ulster University. Participants aged > 50 years were recruited following screening for exclusion criteria (i.e. taking medications known to interfere with B vitamin metabolism, supplements containing B vitamins, consuming > 4 portions of B vitamin-fortified foods per week or diagnosed with gastrointestinal, liver or pulmonary disease). Recruited participants meeting the inclusion criteria were randomised (by sex and study centre) to receive daily for 16 weeks either B vitamin-fortified or placebo drinks as developed by Smartfish, Norway. Each B vitamin-fortified drink (200 ml) contained 200 µg folic acid, 10 µg vitamin B12, 10 mg vitamin B6 and 5 mg riboflavin, while the placebo was an identical, isocaloric formulation without added B vitamins. Fasting blood samples were collected pre- and post-intervention which were used to measure the primary outcome of change in B vitamin biomarker levels.

**Results:**

A total of 95 participants were randomised, of which 81 commenced the trial. Of these, 70 completed (37 in the active and 33 in the placebo groups). Intention to treat (ITT) analysis of the B vitamins demonstrated a significant improvement in all B vitamin biomarkers in the active compared to placebo groups: p < 0.01 for each of serum folate, serum vitamin B12 and plasma pyridoxal 5′-phosphate (vitamin B6) and the functional riboflavin biomarker, erythrocyte glutathione reductase activation coefficient (EGRac). Correspondingly, a significant lowering of serum homocysteine from 11.9 (10.3–15.1) µmol/L to 10.6 (9.4–13.0) µmol/L was observed in response to the active treatment (P < 0.001). Similar results were seen in a per-protocol analysis.

**Conclusions:**

The results demonstrate that a B vitamin-fortified drink was effective in optimising B vitamin status, making this a useful intervention option to improve B vitamin status in older adults.

*Trial registration* ISRCTN, ISRCTN61709781—Retrospectively registered, https://www.isrctn.com/ISRCTN61709781

**Supplementary Information:**

The online version contains supplementary material available at 10.1186/s12986-021-00630-8.

## Background

One-carbon metabolism is a series of interlinking metabolic pathways that provides one-carbon units for the synthesis of DNA, amino acids and phospholipids. The B-vitamins folate, vitamin B12, vitamin B6, and riboflavin are central to the functioning of one-carbon metabolism. A high proportion of older Irish adults are reported to have sub-optimal B vitamin status. Findings from the most recent National Adult Nutrition Survey (NANS) indicated that > 50% of older adults have riboflavin status considered to be low/deficient (EGRac (erythrocyte glutathione reductase activation coefficient) > 1.3), while approximately 20% have insufficient intakes of riboflavin [13]. Results from the Irish Longitudinal Study on Ageing (TILDA) have additionally indicated that 12% and 15% of older adults have low vitamin B12 and folate status, respectively [16]. Furthermore, the prevalence of insufficient folate and vitamin B12 status is much higher among non-consumers of fortified foods, predominantly breakfast cereals and fat spreads, compared to regular consumers [12]. Therefore, new strategies, including novel foods targeted at older people, are needed to improve B vitamin status.

Fortified drinks offer a potential route for enhancing vitamin status, but relatively few studies have investigated the potential of drinks fortified with B vitamins to improve B vitamin biomarker status. The majority of previous studies that investigated the effect of fortified drinks on status were performed in children and have generally included multiple vitamins and minerals to improve nutritional status in geographical areas where micronutrient deficiencies are common [2, 3, 28]. In one such study, children in India received daily a drink fortified with 0.63 mg riboflavin, 35 µg folic acid and 1.27 µg vitamin B12 for 8 weeks [28], resulting in significant improvements in blood concentrations of folate and vitamin B12. Other studies that administered fortified drinks in children focussed on iron supplementation for prevention of anaemia [2, 3]. A limited number of studies have investigated the effect of intervention using B vitamin-fortified drinks in older adults. In one such intervention study using a drink enriched with multiple minerals and vitamins which included 1.9 mg vitamin B2, 2.5 mg vitamin B6, 480 µg folic acid and 5.3 µg vitamin B12 was administered to frail institutionalised elderly people [29]. Following 6 months of daily consumption of the drinks, plasma B12 increased while plasma homocysteine significantly decreased; correspondingly, cognition improved as assessed by scores in the Word Learning test and Category Fluency tests in the group that received the enriched drinks. No results for folate, vitamin B6 or riboflavin biomarkers were reported. A second study used the same multinutrient drink in a larger cohort to investigate the effects of these vitamins on both mental and physical function [20]. Again, the enriched drinks were effective in decreasing homocysteine concentrations and increasing serum vitamin B12, along with vitamin B6 and folate concentrations, but results for riboflavin were not reported. Overall, these studies highlight the potential of drinks fortified in B vitamins as a practical means of enhancing B vitamin status in older adults. Therefore, the objective of this research was to investigate the effectiveness of a novel drink product, fortified with folic acid, vitamin B12, vitamin B6 and riboflavin, in optimising B vitamin status in healthy older adults.

## Materials and methods

### Inclusion criteria and randomisation

The Opti-Age Food Intervention Study was a dual-centre study based in University College Dublin (UCD) and Ulster University, Coleraine (UU) and was approved at each centre by their respective ethics committees (UCD ethics number: LS-18-60-Brennan, UU: REC/18/0033). Free-living adults aged 50 years and over were recruited for this study. The sample size required for this study was estimated using plasma homocysteine as a functional indicator of B-vitamin adequacy and based on the significant difference in plasma homocysteine concentrations (of 2.1 µmol/l) between high consumers and non-consumers of B vitamin fortified foods, as determined from our previous study [11]. Using the online sample size calculator (SPH Analytics [26]), and mean (SD) homocysteine values for non-consumers and high consumers of fortified food of 12.2 (3.9) and 10.1 (3.5), respectively, from our previous study [11], a sample size of 38 subjects per treatment group was estimated based on a power of 80% (β) and a significance level (α) of 0.05. Note in our previous report, the homocysteine values were presented as median and and interquartile range [11], and therefore required conversion to mean (SD) for the purposes of applying the online sample size calculator in the current study. Allowing for potential drop-outs, we aimed to recruit 45 participants per treatment group.

Participants were recruited through posters displayed around each university campus and surrounding areas, notices in local church newsletters, by oral presentation to parents in a local primary school and contacting community groups. Interested individuals were provided with the participant information sheet and allowed time to consider taking part. Potential participants were screened for eligibility using a short interview for fortified food and supplement consumption. Participants were included if they consumed four portions or less of fortified food per week and did not currently or had not in the previous 4 months consumed a supplement containing B vitamins. Participants were excluded if they had a diagnosis of coeliac disease, Crohn’s disease, ulcerative colitis, liver disease (NAFLD and hepatitis) or chronic obstructive pulmonary disease or were taking medications known to interfere with B vitamin metabolism, or if they were taking part in other research or unable to give informed consent to take part. Additionally, women were excluded if they were taking hormone replacement therapy, were pregnant or lactating.

The fortified drinks used in the intervention were manufactured by Smartfish (Norway) and shipped to the two study centres where they were stored at room temperature until distributed. Each 200 ml drink was mixed fruit-flavoured and manufactured to contain 200 µg folic acid, 10 µg vitamin B12, 10 mg vitamin B6 and 5 mg riboflavin in the active version, while the placebo was an identical, isocaloric formulation which did not contain B vitamins. Both the intervention and placebo drinks contained 10 µg vitamin D. B vitamin content in the drinks were independently analysed after manufacture and analysis was repeated during and post intervention. The drinks were provided in opaque cartons. The intervention was double blinded with an independent researcher at each centre responsible for packing the drinks into opaque bags for distribution. Eligible participants were randomised, with gender and centre as factors in the randomisation, by an independent researcher to receive either the B vitamin or placebo version of the drinks to consume once daily for 16 weeks.

### Data collection

Participants attended a study centre for data collection pre and post intervention. At each appointment, anthropometric measurements (height, weight, waist and hip circumference) and blood pressure (following NICE guidelines) were measured by centrally trained researchers and a short previously used questionnaire on health, diet and lifestyle was administered [17, 23].

Blood pressure was measured using an Omron 705IT monitor. One measurement was taken initially from each arm. The arm from which the highest measurement was obtained was then considered the reference arm. Measurements were then taken from the reference arm at 1-min intervals until two measurements which agreed to within 5 mmHg for both systolic and diastolic blood pressure were obtained, and the mean of these was calculated.

A fasting blood sample was also collected at each appointment into the following tubes: 1 × 8 ml serum, 1 × 6 ml lithium heparin, 2 × 9 ml EDTA (one immediately put on ice, the other kept at room temperature). Serum tubes were left to clot for 30 min prior to centrifugation. Lithium heparin samples were centrifuged at 1800×*g* for 10 min at 4 °C and aliquoted within 1 h of the sample being drawn. Serum and EDTA samples were centrifuged at 1628×*g* for 15 min at 4 °C and the supernatant subsequently aliquoted. In EDTA tubes, the buffy coat was removed before the red blood cells were washed 3 times with phosphate-buffered saline. After each centrifugation, the saline was removed and washed red cells were aliquoted within a maximum of 4 h of sample draw. All aliquots were stored at − 80 °C until analysis. Serum samples were used to analyse for homocysteine and biomarkers of vitamin D, vitamin B12 and folate. EDTA plasma samples were used to analyse vitamin B6 biomarkers and EDTA washed red blood cells were used to analyse a biomarker of riboflavin status.

### Delivery of the intervention and assessment of compliance

Prior to commencing the intervention, participants completed a 4-day food diary. This diary was examined by researchers and based on the contents, participants were advised of substitutions and alterations that could be made to their diet to account for the daily addition of the approximately 200 kcal drink. Participants were advised to reduce consumption of foods such as confectionary, crisps, desserts, biscuits and chocolate, i.e. foods high in calories but with limited nutritional value. A dietary advice sheet was also provided to participants to refer to during the study. Participants generally received their drinks in 2–3 lots for ease of transport. Compliance was assessed using a checklist which participants were instructed to complete daily on consumption of a drink. In addition, any drinks not consumed by participants during the 16-week intervention period were collected and counted. Of the 70 participants who completed the study, 59 had compliance > 90%, a further 7 participants had compliance of 80–90%, while only one participant consumed < 80% of the drinks (compliance data was missing for n = 3 participants).

### Biomarker analysis

Riboflavin status was determined at Ulster University using an erythrocyte glutathione reductase activation coefficient assay (EGRac) which measures the activity of the enzyme glutathione reductase before and after in vitro reactivation with its prosthetic group FAD. EGRac is calculated as the ratio of FAD-stimulated to unstimulated enzyme activity, with a value ≥ 1.30 generally indicative of suboptimal riboflavin status, while values ≥ 1.40 are considered to indicate riboflavin deficiency. Plasma pyridoxal 5′-phosphate (PLP) concentration served as a biomarker of vitamin B-6 status and was measured, also at ulster University, by reversed phase, high performance liquid chromatography with fluorescence detection [4]. Serum folate, vitamin B12 and homocysteine were measured at Bevital Laboratory, Bergen, Norway (www.bevital.no). Serum folate concentrations were measured by a microbiological assay based on a chloramphenicol-resistant strain of *Lactobacillus casei* [21]. Value below the WHO cut-off of 10 nmol/L were considered to indicate deficiency. Serum vitamin B12 was determined by microbiological assay using *Lactobacillus leichmanni*, while homocysteine was measured by fluorescence polarisation assay. Serum vitamin B12 concentrations below 150 pmol/L were considered deficient, values of 150–221 pmol/L were indicative of insufficiency while values above 221 pmol/L were considered sufficient. For homocysteine, the recent consensus statement on homocysteine and dementia cut-off was used, with values above 11 nmol/L indicating elevated homocysteine. Serum vitamin D was measured on an API 4000 AB Sciex liquid chromatography mass spectrometer (AB Sciex UK Limited, Warrington, UK) as 25-hydroxyvitamin D (the recognised blood marker of vitamin D status). A proprietary assay method developed by Chromsystems GmbH, Munich, Germany (MassChrom® 25-OH-Vitamin D3/D2 in Serum/Plasma) was used and samples underwent a pre-assay extraction followed by further purification by high pressure liquid chromatography before injection into the mass spectrometer. Vitamin D biomarker analysis was conducted in St James’ Hospital, Dublin.

### Statistical analysis

All biomarker data were inspected for extreme outliers (> 2 standard deviations from the mean). One such outlier was identified for folate at the pre-intervention timepoint and was removed from the database. Imputation of this and other missing values was performed by a statistician using 3 different methods – Random Forrest imputation, Bayesian linear regression imputation and Bayesian imputation with bootstrapping. The most appropriate method for each biomarker was deemed to be the one resulting in the lowest error values and were as follows: Bayesian linear regression for total vitamin D, homocysteine and vitamin B12; Random Forrest for riboflavin (measured by EGRac); and Bayesian with bootstrapping for folate. The values obtained were entered into the final version of the databank prior to analysis.

Statistical analysis was performed using SPSS v24. Intention-to-treat analysis was performed, followed by a per-protocol analysis where only data from participants who completed the 16-week intervention were included. Normality tests were performed, and all non-normally distributed data were log-transformed prior to analysis. Differences between intervention and placebo group at baseline were assessed using independent samples t-tests, as well as the difference between centres. A baseline-adjusted Analysis of Covariance (ANCOVA) model was fitted for each outcome with study group as a factor. The ANCOVA models for B vitamin biomarkers were then further adjusted for baseline homocysteine status.

## Results

### Monitoring of B vitamin stability in the fortified drinks

Characterisation of the content of the drinks was performed 3 times over the course of 19 months. The active drink was identical to placebo in terms of caloric and nutrient composition with the exception of B vitamin content (Table 1). One month following manufacture all four B vitamins were present at the intended concentration. Following a shelf life of 14 months, vitamin B2, vitamin B6 and vitamin B12 remained stable. However, a decline in folic acid content was detected (90 μg remaining—55% loss of folic acid). Further analysis at 19 months post manufacture revealed that folic acid had declined further to 56 µg (72% of folic acid lost). At this timepoint there was no decline in vitamin B2, B6 or B12 (Table 2).

**Table 1:** Nutritional content (per 200 ml portion) of the placebo drinks and active drinks

	Placebo	Active
Energy (kcal)	211	211
Protein (g)	6.4	6.4
Carbohydrate (g)	21.7	21.7
Fat (g)	11	11
Omega 3 (mg)	2400	2400
Vitamin D3 (µg)	10	10
Folic acid (µg)	0	200
Vitamin B12 (µg)	0	10
Vitamin B6 (mg)	0	10
Riboflavin (mg)	0	5

Nutrients reported as concentration per 200 ml drink; participants received 1 × 200 ml portion daily

**Table 2:** B vitamin content of the drinks throughout the intervention period

Time after manufacture	Folic acid (µg)	Vitamin B12 (µg)	Vitamin B6 (mg)	Riboflavin (mg)
1 month	200	10	10	5
14 months	90	12	10	5
19 months	56	15	14	6

B vitamins reported as concentration per 200 ml drink; participants received 1 × 200 ml portion daily

### Supplementation with B vitamin drinks for 16 weeks improved B-vitamin status

**Fig. 1:**
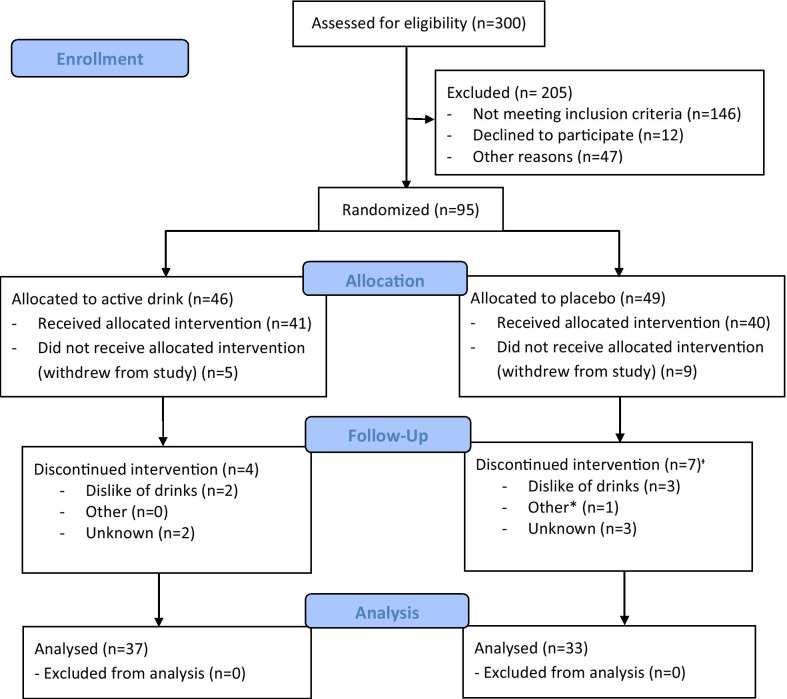
CONSORT flow diagram for opti-age food intervention study. *Participant discontinued taking the drinks as they travelled abroad frequently and were unable to take drinks with them. +One participant who discontinued taking the drinks provided a blood sample at a debriefing appointment which was included in the intention-to-treat analysis

A total of 81 participants commenced the study, 37 completed the active arm and 33 completed the placebo arm (Fig. 1). Participants had a mean age of 64.9 years, and median body mass index (BMI) of 25.4 kg/m^2^ (IQR 23.41–29.46). Median riboflavin status (EGRac of 1.31) was in the insufficient range, with 54.3% of participants having EGRac values indicating riboflavin deficiency or insufficiency (EGRac ≥ 1.3). Median homocysteine concentrations were relatively high (11.55 nmol/L), with 59.3% of participants’ homocysteine above the recent consensus statement on homocysteine and dementia cut-off of 11 nmol/L. All participants had serum vitamin B6 concentrations above the commonly used cut-off for deficiency of 20 nmol/L, while a small number (4.9%) had serum concentrations considered suboptimal (20–30 nmol/L). Most participants had sufficient serum folate (91.4% above World Health Organisation cut-off of 10 nmol/L) and vitamin B12 (81.5% of participants with normal B12 concentrations i.e. above 221 pmol/L) status.

The active and placebo groups were matched in terms of anthropometric variables and B vitamin status. Homocysteine concentrations, however, were borderline significantly higher at baseline in the intervention group compared with the control group (p = 0.047) (Table 3). Differences between study centres were also examined, with no significant difference in baseline of any of the B vitamin biomarkers. UCD participants were significantly younger, had lower waist circumference and blood pressure and had significantly higher vitamin D concentrations compared to participants at the UU centre.

**Table 3:** Baseline characteristics of treatment groups

Demographic variable	Group		P value
	Placebo	Active	
	(*n* 40)	(*n* 41)	–
Age (years)	65.2 (8.4)	64.7 (6.3)	0.784
Sex *n* male (female)	17 (23)	16 (25)	–
Weight (kg)	73.0 (63.4–84.0)	72.8 (63.3–84.2)	0.916
Height (m)	1.69 (0.09)	1.68 (0.08)	0.587
BMI (kg/m^2^)	25.5 (23.5–29.5)	25.4 (23.4–29.5)	0.860
Waist (cm)	92.70 (82.63–102.50)	89.75 (81.50–105.05)	0.702
Hip (cm)	104.28 (8.56)	104.42 (8.74)	0.944
Waist:hip	0.89 (0.09)	0.90 (0.10)	0.605
Systolic BP (mmHg)	130.9 (19.7)	127.6 (20.3)	0.469
Diastolic BP (mmHg)	76.2 (8.6)	74.6 (10.1)	0.321
Plasma total 25(OH)D (nmol/L)	62.5 (22.4)	59.3 (25.2)	0.539
Serum Homocysteine (µmol/L)	11.05 (9.68–12.75)	11.86 (10.29–15.05)	0.047
Serum folate (nmol/L)	18.0 (14.3–21.9)	15.5 (11.8–21.6)	0.123
Serum vitamin B12 (pmol/L)	336 (235–449)	310 (246–409)	0.357
Plasma PLP (vitamin B6; nmol/L)	61.7 (48.6–85.1)	59.4 (44.5–74.1)	0.487
Riboflavin (EGRac)	1.29 (1.24–1.35)	1.34 (1.25–1.43)	0.168

Data presented as mean (SD) and median (IQR). P value of differences between treatment groups, p < 0.05 considered statistically significant. BP, blood pressure; EGRac, erythrocyte glutathione reductase activation coefficient; PLP, Pyridoxal 5′-phosphate

Intention to treat (ITT) analysis of the B vitamins demonstrated a significant improvement in status of all measured B vitamins post intervention in response to the fortified drinks. Furthermore, a significant lowering of homocysteine concentrations from 11.86 to 10.56 µmol/L was observed (Table 4). Vitamin D concentrations did not differ between the groups following the intervention (Table 4). Controlling for baseline homocysteine concentrations did not impact the results except for vitamin B6; following inclusion of homocysteine in the model the change in vitamin B6 was no longer significant (p = 0.075) (Additional file 1: Table S1).

**Table 4:** B vitamin biomarkers before and after intervention with a B vitamin-fortified and placebo drink

	Placebo		Active		P value
	Pre-intervention	Post-intervention	Pre-intervention	Post-intervention	
Plasma Total 25(OH)D (nmol/L)	62.5 (22.4)	66.9 (17.0)	59.3 (25.20)	65.5 (25.8)	0.727
Serum homocysteine (µmol/L)	11.1 (9.7–12.8)	11.3 (9.9–13.2)	11.9 (10.3–15.1)	10.6 (9.4–13.0)	0.000240
Serum folate (nmol/L)	18.0 (14.3–22.0)	16.6 (13.0–21.8)	15.5 (11.8–21.6)	17.9 (12.7–23.6)	0.000439
Serum vitamin B12 (pmol/L)	336 (235–449)	342 (268–468)	310 (246–409)	428 (307–549)	0.005
Plasma PLP (vitamin B6; (nmol/L)	61.7 (48.6–85.1)	65.4 (38.3–104.9)	59.4 (44.5–74.1)	253.0 (181.0–351.4)	0.002
Riboflavin (EGRac)	1.29 (1.24–1.35)	1.30 (1.23–1.36)	1.34 (1.25–1.43)	1.14 (1.09–1.23)	3.2014 × 10^–9^

Data presented as mean (SD) or median (IQR). P value of group effects when analysed by ANCOVA adjusted for baseline values in intention-to-treat analysis. P < 0.05 considered statistically significant. EGRac, erythrocyte glutathione reductase activation coefficient; PLP, Pyridoxal 5′-phosphate

Inclusion of only participants who completed the 16-week intervention in a per-protocol analysis yielded similar results. The baseline characteristics were similar with baseline homocysteine concentrations significantly different between the groups (p = 0.035) (Table 5). The intervention significantly improved all B vitamin biomarkers compared to placebo (Additional file 1: Table S2). All changes in the B vitamin remained significant following adjustment for baseline homocysteine concentrations (Additional file 1: Table S3). Per-protocol analysis performed for anthropometric variables (weight, BMI, systolic and diastolic blood pressure) found no significant differences between the active and placebo group after the 16-week intervention (Table 6).

**Table 5:** Baseline characteristics of the treatment groups (per-protocol)

Baseline characteristic	Group		P value
	Placebo	Active	
	(*n* 33)	(*n* 37)	–
Age (years)	64.6 (8.2)	64.4 (6.0)	0.892
Sex (% female)	54.5	59.5	–
Weight (kg)	72.5 (61.7–80.2)	72.8 (63.3–84.2)	0.881
Height (m)	1.69 (0.10)	1.68 (0.08)	0.535
BMI (kg/m^2^)	24.8 (23.0–28.3)	25.4 (23.4–29.5)	0.582
Waist (cm)	90.8 (81.5–100.3)	89.8 (81.5–105.1)	0.539
Hip (cm)	102.0 (97.8–107.3)	102.1 (99.6–109.1)	0.844
Waist:hip	0.88 (0.09)	0.90 (0.10)	0.455
Systolic BP (mmHg)	129.2 (20.0)	127.2 (21.1)	0.694
Diastolic BP (mmHg)	76.0 (9.2)	75.2 (11.5)	0.765
Plasma total 25(OH)D (nmol/L)	59.6 (49.3–73.5)	58.8 (37.6–65.4)	0.500
Serum homocysteine (µmol/L)	10.5 (9.6–12.6)	11.6 (10.0–14.8)	0.035
Serum folate (nmol/L)	18.2 (14.1–24.0)	15.5 (12.5–21.6)	0.177
Serum vitamin B12 (pmol/L)	344 (243–439)	310 (246–413)	0.374
Plasma PLP (vitamin B6 (nmol/L)	63.9 (49.1–79.8)	59.4 (45.5–79.4)	0.388
Riboflavin (EGRac)	1.28 (1.22–1.34)	1.34 (1.25–1.41)	0.088

Data presented as mean (SD) and median (IQR). Per-protocol analysis includes all participants who completed the 16-week intervention. P value of differences between groups, p < 0.05 considered statistically significant. BP, blood pressure; EGRac, erythrocyte glutathione reductase activation coefficient; PLP, Pyridoxal 5′-phosphate

**Table 6:** Anthropometric and blood pressure before and after a 16-week intervention with B vitamin-fortified drinks (per-protocol analysis)

	Placebo		Active		P value
	Pre-intervention	Post-intervention	Pre-intervention	Post-intervention	
Weight (kg)	72.5 (61.7–80.2)	74.5 (63.0–83.0)	72.8 (63.3–84.2)	74.4 (64.5–83.8)	0.593
BMI (kg/m^2^)	24.8 (23.0–28.3)	24.9 (23.6–29.1)	25.4 (23.4–29.5)	25.2 (23.6–29.3)	0.618
Waist circumference (cm)	90.8 (81.5–100.3)	93.0 (82.0–100.0)	89.8 (81.5–105.1)	89.0 (84.5–107.0)	0.967
Systolic BP (mmHg)	129.2 (20.0)	130.9 (16.3)	127.2 (21.1)	127.7 (19.2)	0.516
Diastolic BP (mmHg)	76.0 (9.2)	76.2 (8.6)	75.2 (11.5)	74.6 (10.1)	0.503

Data presented as mean (SD) and median (IQR). Per-protocol analysis includes participants who completed the 16-week intervention. P value of group effects when analysed by ANCOVA adjusted for baseline status. P < 0.05 considered statistically significant. BP, blood pressure

## Discussion

The results of the 16-week intervention demonstrated that the B vitamin-fortified drinks were a successful vehicle for optimising B vitamin status in older adults. Compared with placebo, there was a significant increase in the concentration of folate, vitamin B12, vitamin B6 and as well as a significant decrease in the related metabolite, homocysteine. The four B vitamins included in the fortified drinks are involved in one-carbon metabolism and it is well established that supplementation with these B vitamins can lower homocysteine levels [7].

To our knowledge, there are limited interventions using a fortified drink to deliver B vitamin supplementation in an older population. A previous study delivered multiple vitamins, including B vitamins and minerals in a drink for 6 months to an older population [29]. Similar results to the present study were obtained for vitamin B12 with an improvement in concentrations while homocysteine concentrations were also significantly decreased. Interestingly the B vitamin content of the drinks were lower than our study indicating the effectiveness of low-dose B vitamin supplementation. In a second study using the same concentrations of B vitamins in fortified drinks as the current study, improvements in vitamin B6 and folate status after 24 weeks of supplementation was reported [20]. Collectively, the results demonstrate the potential of fortified drinks as viable options for improvement of B vitamin status. Furthermore, the wide age range (51–84 years) of the participants included support the potential of such drinks in promoting healthy ageing.

To date the majority of work concerning optimisation of B vitamin status has focused on the use of capsules in supplementation studies. However, comparing the results of our intervention to B vitamin interventions using capsules is more difficult as capsule interventions are generally of much longer duration and intervene with higher amounts of B vitamins. For example, the VITACOG study supplemented participants for 2 years with 800 µg folic acid, 500 µg vitamin B12 and 20 mg vitamin B6 daily [27]—4 times the amount of folic acid and 50 times the amount of vitamin B12 used in the Opti-Age Food Intervention Study. As a result, plasma folate concentrations increased by 270% and plasma vitamin B12 doubled in VITACOG participants. In comparison, Opti-Age participants consuming the active drinks had a mean serum folate increase of 16% and a mean serum vitamin B12 increase of 41%. Given the shorter intervention and much lower amount of vitamin B12 in the drinks compared to the VITACOG supplement, the Opti-Age Food Intervention Study was very effective in increasing vitamin B12 status. The lower percent change in serum folate was likely due to the decomposition of folic acid in the Opti-Age drinks over time. A supplement in tablet form containing 1 mg of vitamin B12 was given to participants with vitamin B12 deficiency for 1 year, which caused a 177% increase in the vitamin B12 concentrations of participants [6]. Here, again, the higher dose and longer duration of this study likely accounts for the size of the increase in vitamin B12 concentrations. An Australian study which provided B vitamin supplementation in the form of a multivitamin, mineral and antioxidant tablet had an intervention of the same duration as the Opti-Age Food Intervention Study—16 weeks [10]. This supplement had a men’s and women’s version with the men’s version containing 35 mg riboflavin, 25 mg vitamin B6, 500 µg folic acid and 120 µg vitamin B12 and the women’s version containing 30 mg riboflavin, 30 mg vitamin B6, 500 µg folic acid and 115 µg vitamin B12. However, a significant effect of supplementation was only seen for vitamin B6 and B12, with no effect on riboflavin or folate status. Although comparison of the two methods of supplementation is difficult, and there are many factors that may also impact the success of interventions such as the baseline B vitamin status, the form of the B vitamins used (e.g. folic acid vs methyltetrahydrofolate) and the age and health status of the participants, the present results indicate that improvements in B vitamin status can be achieved using lower concentrations of B vitamins in a drink format. Supplementation with lower concentrations of B vitamins may be preferable as an amount more in line with daily recommendations and be more readily accepted by consumers. In addition, using low-dose supplementation greatly reduces the risk of B vitamin toxicity or overconsumption. A Tolerable Upper Limit of 25 mg/day has been set for vitamin B6 intake in Ireland while a limit of 1 mg/day has been adopted for folic acid as high intakes have the potential to mask vitamin B12 deficiency [8]. In the case of vitamin B12, no upper limit has been set as bioavailability is low. Although prevalence of insufficient levels of B6, B12 and folate were lower in our study than previously observed, we found high prevalence of insufficiency in riboflavin and higher than recommended homocysteine levels, the latter of which can be affected by all B vitamins included in the drinks. Therefore, the drinks can be useful to optimize B vitamin status and associated metabolites in older populations.

Despite a policy of voluntary fortification of foods, it is clear from previous evidence that a significant proportion of older adults living in Ireland have sub-optimal B vitamin status. It is also evident that the current policy of voluntary fortification leaves a significant proportion of the population vulnerable to low intakes of B vitamins, with results from NANS identifying over 20% of Irish adults as being non-consumers of foods fortified with folic acid and over 30% as non-consumers of foods fortified with vitamin B12 [12]. In addition to this, only 16% of non-consumers of fortified food had optimal red blood cell folate status compared with 47% of those classed as high consumers of fortified food. In the TUDA cohort, red blood cell concentrations of folate significantly increased with increasing levels of fortified food consumption [22]. This is consistent with previous evidence from Northern Ireland which showed that fortified food intake was associated with significantly higher intakes and biomarker status of B vitamins [11]. Therefore, fortified foods have an essential role to play in optimising B vitamin intakes and biomarkers in older adults.

Within the present work we observed a sharp decline in folic acid concentrations following manufacture. The drinks used in our intervention had fruit juice as the main ingredient. A previous study examining the decline of folic acid content in various fruit juice drinks over time found an average 46% decrease in folic acid content by the end of the testing period [9]. The drink products tested initially contained 90% more folic acid than indicated on the label but this had decreased to around, or just below, the concentration indicated after 1 year. Therefore, adding more folate can be a strategy to deal with degradation over time, but large amounts are needed to ensure that concentrations are still above the target after long term storage. Numerous factors can affect the stability of folic acid in drinks, such as the pH of the solution with folic acid showing reduced stability at acidic pH values [1]. As the pH of the Opti-Age drinks was between 3.5 and 4, this was most likely a factor contributing to the decline in the folic acid concentration. Other factors such as light also cause accelerated degradation of folic acid [9]; however, the Opti-Age drinks were packed in opaque cartons to reduce this impact. Additionally, research has shown that small amounts of ethanol, present in added flavourings can also attenuate folic acid stability in drinks when combined with an acidic pH [14]. Therefore, future research into strategies such as encapsulation is needed to optimise the incorporation of folic acid into fortified fruit drinks [25]. Despite the decline in folic acid content, a significant effect of consuming the B vitamin-fortified drinks on folate status was observed.

Both the intervention and placebo drinks contained 10 µg vitamin D per drink. Although there was a significant difference in vitamin D status between study centres at baseline, there was no difference between the intervention and placebo group. Additionally, there was no treatment effect of vitamin D, as expected. In general, vitamin D status in this population was optimal prior to the intervention starting with 70.4% of participants having a serum vitamin D concentration of > 50 nmol/L. Only 3.7% of participants had a baseline serum vitamin D concentration of < 25 nmol/L. However, supplementation with vitamin D contained in fortified drinks appeared to prevent a trough in vitamin D concentrations that can often occur in the winter and spring months at higher latitudes [18, 19]. The difference between centres at baseline can perhaps be explained by the difference in latitude between Dublin (UCD) and Coleraine (UU), and also by the fact that UCD participants commenced the intervention earlier in the year compared to the UU participants (August–October compared to October–December). While numerous previous intervention studies have used Smartfish drinks containing vitamin D, vitamin D status was generally not a primary outcome and pre- and post-intervention vitamin D status was not reported [5, 15, 24]. However, our results clearly show maintenance of vitamin D status was achieved over the winter months with daily consumption of a drink containing 10 µg of vitamin D. The present drinks contain 200 kcal per drink and any future use should take suitable steps to substitute the drinks for unhealthy snacks or other foods to avoid and increase in energy intake.

One limitation of this study was the loss of folic acid in the drinks over time following manufacture. Therefore, although we showed a significant increase in serum folate concentrations after 16 weeks of intervention with the fortified drink, this response may have been less than expected owing to participants receiving less folic acid per drink than intended. Likewise, the corresponding decrease in homocysteine concentrations as a result of intervention may have been lessened. Another limitation is that we relied on the participants to self-report their compliance and to return any drinks that were not consumed. To combat this an ITT analysis was performed. Also, as a result of a higher than expected drop-out rate in the placebo group, the total of number of participants who completed the trial (i.e. 37 in the active arm and 33 in the placebo arm) was somewhat less than our sample size estimate of 38 subjects required per treatment group. However, we do not consider this to be a serious limitation given the current results which, despite the smaller participant numbers, showed significant increases in the status of all B vitamins, and correspondingly, a decrease in the related metabolite homocysteine, after intervention with the fortified drink. The major strength of the study is that we used a randomised, double-blinded study design, providing cause-and-effect evidence that the improvement in B vitamin status shown is owing to the B vitamins provided by the fortified drinks, rather than any other dietary factors. The addition of vitamin D to both the active and placebo drinks was another strength of the study, as it benefitted all participants, regardless of group assignment and therefore encouraged compliance. The statistical approach used is another strength of the research. Missing biomarker data were imputed using three methods with the most appropriate for each biomarker chosen allowing all participants who commenced the intervention to be included in analysis with realistic post-intervention data.

## Conclusions

In conclusion, a B vitamin-fortified drink was effective in optimising B vitamin status and was generally well received by participants, making this a useful intervention strategy to improve B vitamin status in older adults. Further studies should address whether there are longer term health benefits for older adults of intervention with folate and related B vitamins at these intake levels.

## Supplementary Information

**Additional file 1: Table S1**. Impact of B vitamin drinks on B vitamin biomarkers controlling for baseline homocysteine concentrations. **Table S2**. B vitamin biomarkers at baseline and end of intervention with the drinks (Per protocol analysis). **Table S3**. Impact of B vitamin drinks on biomarker status controlling for baseline homocysteine concentrations (per-protocol analysis).

## Data Availability

The datasets used during the current study are available from the corresponding author on reasonable request.

## Abbreviations

NANS: National Adult Nutrition Survey
TILDA: The Irish Longitudinal Study on Ageing
EGRac: Erythrocyte glutathione reductase activation coefficient
UCD: University College Dublin
UU: Ulster University
NAFLD: Non-alcoholic fatty liver disease
NICE: National Institute for Health and Care Excellence
EDTA: Ethylenediaminetetraacetic acid
FAD: Flavin adenine dinucleotide
ANCOVA: Analysis of co-variance
BMI: Body Mass Index
ITT: Intention to treat
