# Supplementary material for: Effectiveness of a fortified drink in improving B vitamin biomarkers in older adults: a controlled intervention trial
Source: Nutr Metab (Lond). 2021 Dec 7;18:104. doi: 10.1186/s12986-021-00630-8 (PMC8650259; doi:10.1186/s12986-021-00630-8)
Supplement: Supplementary file 1 — Additional file 1: Table S1. Impact of B vitamin drinks on B vitamin biomarkers controlling for baseline homocysteine concentrations. Table S2. B vitamin biomarkers at baseline and end of intervention with the drinks (Per protocol analysis). Table S3. Impact of B vitamin drinks on biomarker status controlling for baseline homocysteine concentrations (per-protocol analysis). [file 12986_2021_630_MOESM1_ESM.docx]

**Supplementary table 1**: Impact of B vitamin drinks on B vitamin biomarkers controlling for baseline homocysteine concentrations.

|  | **Placebo** | | **Active** | | **P value** |
| --- | --- | --- | --- | --- | --- |
| **Vitamin biomarker** | **Pre-intervention** | **Post-intervention** | **Pre-intervention** | **Post-intervention** |  |
| Folate (nmol/L) | 18.0(14.3-21.9) | 16.6(13.0-21.8) | 15.5(11.8-21.6) | 17.9(12.7-23.6) | 0.000421 |
| Vitamin B12 (pmol/L) | 336(235-449) | 342(268-468) | 310(246-409) | 428(307-549) | 0.003 |
| Vitamin B6 (nmol/L) | 61.7(48.6-85.1) | 65.4(38.3-104.9) | 59.4(44.5-74.1) | 253.0(181.0-351.4) | 0.075 |
| Riboflavin (EGRac) | 1.29(1.24-1.35) | 1.30(1.23-1.36) | 1.34(1.25-1.43) | 1.14(1.09-1.23) | 1.0847x10^-8^ |

Data presented as median(IQR). P value of group effects when analysed by ANCOVA adjusted for baseline status and baseline homocysteine status in intention-to-treat analysis. P<0.05 considered statistically significant. EGRac; erythrocyte glutathione reductase activation coefficient.

**Supplementary Table 2:** B vitamin biomarkers at baseline and end of intervention with the drinks (Per protocol analysis)

|  | **Placebo** | | **Active** | |  |
| --- | --- | --- | --- | --- | --- |
| **Vitamin biomarker** | **Pre-intervention** | **Post-intervention** | **Pre-intervention** | **Post-intervention** | **P value** |
| Total 25(OH)D (nmol/L) | 59.6(49.3-73.5) | 62.7(56.2-78.4) | 58.8(37.6-65.4) | 62.9(51.0-79.6) | 0.805 |
| Homocysteine (µmol/L) | 10.5(9.6-12.6) | 10.7(9.8-12.7) | 11.6(10.0-14.8) | 10.2(9.3-12.8) | 0.00004 |
| Folate (nmol/L) | 18.2(14.1-24.0) | 15.6(12.7-20.8) | 15.5(12.5-21.6) | 19.2(14.4-26.8) | 0.000071 |
| Vitamin B12 (pmol/L) | 344(243-439) | 347(276-468) | 310(246-413) | 428(330-549) | 2.9407x10^-8^ |
| Vitamin B6 (nmol/L) | 63.9(49.1-79.8) | 57.9(36.6-79.0) | 59.4(45.5-79.4) | 275.6(193.1-370.3) | 0.002 |
| Riboflavin (EGRac) | 1.28(1.22-1.34) | 1.31(1.27-1.36) | 1.34(1.25-1.41) | 1.14(1.09-1.20) | 9.7791x10^-12^ |

Data presented as median(IQR). P value of group effects when analysed by ANCOVA adjusted for baseline status. Per-protocol analysis includes all participants who completed the 16-week intervention. P<0.05 considered statistically significant. EGRac; erythrocyte glutathione reductase activation coefficient.

**Supplementary Table 3**: Impact of B vitamin drinks on biomarker status controlling for baseline homocysteine concentrations (per-protocol analysis).

|  | **Placebo** | | **Active** | |  |
| --- | --- | --- | --- | --- | --- |
| **Vitamin biomarker** | **Pre-intervention** | **Post-intervention** | **Pre-intervention** | **Post-intervention** | **P value** |
| Folate (nmol/L) | 18.2(14.1-24.0) | 15.6(12.7-20.8) | 15.5(12.5-21.6) | 19.2(14.4-26.8) | 0.000090 |
| Vitamin B12 (pmol/L) | 344(243-439) | 347(276-468) | 310(246-413) | 428(330-549) | 4.6123x10^-8^ |
| Vitamin B6 (nmol/L) | 63.9(49.1-79.8) | 57.9(36.6-79.0) | 59.4(45.5-79.4) | 275.6(193.1-370.3) | 0.027 |
| Riboflavin (EGRac) | 1.28(1.22-1.34) | 1.31(1.27-1.36) | 1.34(1.25-1.41) | 1.14(1.09-1.20) | 4.6245x10^-11^ |

Data presented as median(IQR). P value of group effects when analysed by ANCOVA adjusted for baseline status and baseline homocysteine status. Per-protocol analysis includes all participants who completed the 16-week intervention P<0.05 considered statistically significant. EGRac = erythrocyte glutathione reductase activation coefficient.
